# Crucial Roles of the Assistant Surgeon During Laparoscopic Left Hemihepatectomy

**DOI:** 10.7759/cureus.24050

**Published:** 2022-04-11

**Authors:** Masatoshi Kajiwara, Fuminori Ishii, Takahide Sasaki, Ryo Nakashima, Suguru Hasegawa

**Affiliations:** 1 Department of Gastroenterological Surgery, Faculty of Medicine, Fukuoka University, Fukuoka, JPN

**Keywords:** arantius ligament, glissonean approach, assistant surgeon, left hemihepatectomy, laparoscopic hepatectomy

## Abstract

Background

Although left hemihepatectomy has been widely performed via the laparoscopic approach, the roles of the assistant surgeon have not been well-documented so far. We herein present our standardized procedures of laparoscopic left hemihepatectomy without Spiegel’s lobe resection, focusing on the crucial roles of the assistant surgeon.

Methods

During laparoscopic left hemihepatectomy without Spiegel’s lobe resection, countertraction by the assistant surgeon is quite important especially during isolating the left Glissonean pedicle and transecting liver parenchyma. When securing the left hepatic pedicle using the Glissonean approach, the assistant surgeon pushes Segment 4 of the liver cranially and pulls the tape encircling the hepatoduodenal ligament caudally in the opposite way, orthogonal to the direction of the laparoscopic forceps toward the left portal triad. During liver parenchymal transection, the assistant surgeon pulls the hanging tape across the left lobe of the liver in order to provide a wide and stable liver transection plane.

With this standardized technique, nine cases of laparoscopic left hemihepatectomy were performed over the last two years in our department, and the perioperative data were retrospectively analyzed.

Results

The median age of the nine patients was 70 years (range: 58 - 84 years). Most of the patients were males (77.8%). Five of nine patients were diagnosed with colorectal liver metastasis, two with hepatocellular carcinoma (HCC), one with inflammatory pseudotumor, and the other one with hepaticolithiasis. There were no conversions to open surgery. The median operative time and estimated blood loss were 337 minutes (range: 219 - 478 minutes) and 100 ml (range: 41 - 375 ml), respectively. The median length of postoperative hospital stay was nine days (range: 7 - 16 days). Major complications (Clavien-Dindo classification grade III or more) were not encountered in our cohort postoperatively.

Conclusion

We presented here our standardized assistant roles during laparoscopic left hemihepatectomy without Spiegel’s lobe resection, which was revealed to be safe and feasible in our cohort.

## Introduction

Laparoscopic anatomical liver resection has become feasible over the past decade but is still challenging. Among laparoscopic major hepatectomies, left hemihepatectomy without Spiegel’s lobe resection is reported to be suitable as a first step for young surgeons because it does not require dissection around the inferior vena cava (IVC) and its transection plane is single and smaller than that of the other types of major hepatectomies [1]. To date, there are many reports on laparoscopic left hemihepatectomy [1-8]. Although they mainly mentioned standardized surgical procedures or safety and efficacy, they usually did not refer to the definite roles of the assistant surgeon.

In laparoscopic surgery, we usually experience motion restrictions of straight-fixed instruments for obtaining effective surgical angles. To overcome these limitations, optimal countertraction of the assistant surgeon is quite essential. Then, we standardized the surgical procedures of laparoscopic left hemihepatectomy without Spiegel’s lobe resection not only for the main surgeon but for the assistant surgeon also. In order to maximize the effectiveness of assistant support, we used the umbilical tapes to effectively pull the target tissues during isolation of the left Glissonean pedicle and transecting liver parenchyma. The aim of this study was to describe the perioperative outcomes, safety, and feasibility in patients undergoing laparoscopic left hemihepatectomy with our standardized procedures focusing on the assistant surgeon.

## Materials and methods

Patients and methods

From January 2020 to March 2022, nine cases underwent laparoscopic left hemihepatectomy without Spiegel’s lobe resection at Fukuoka University Hospital, Japan. Consent was obtained from all participants in this study. Ethics approval and IRB number were not obtained, as it is a small case series of only nine cases.

Perioperative parameters, including patient’s age, gender, body mass index (BMI), pathologic condition, operative time, estimated blood loss, and length of postoperative hospital stay, were obtained from the medical records retrospectively. All continuous variables were expressed as median with range. Postoperative complications were expressed using the Clavien-Dindo classification [9].

Surgical technique

**Figure 1:**
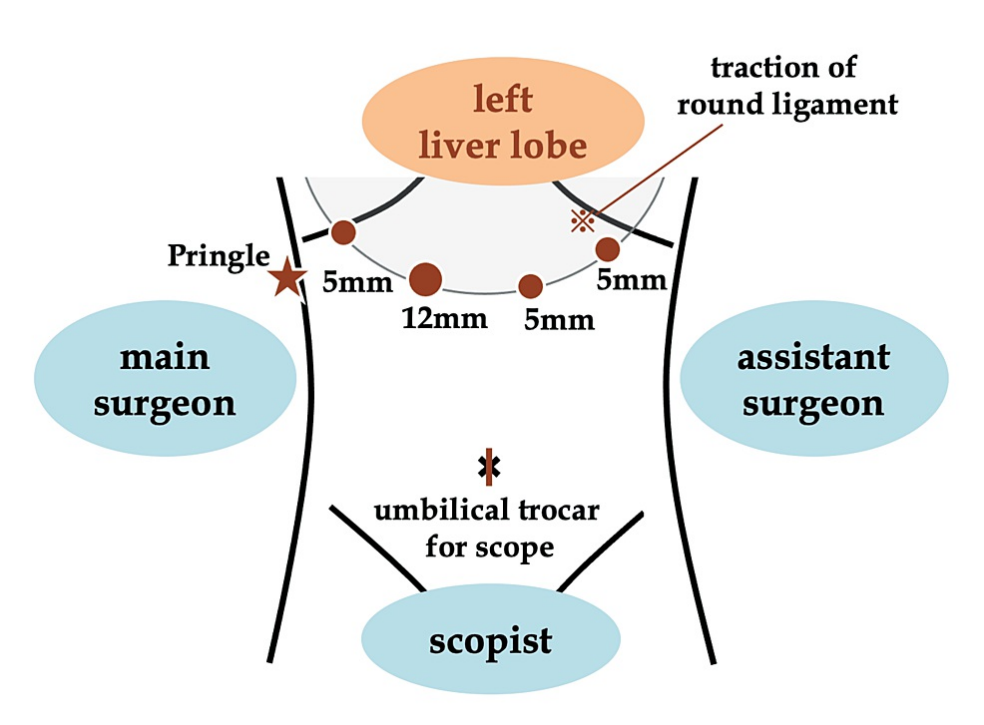
Trocar placement for laparoscopic left hemihepatectomy Four operating trocars (one 12-mm trocar and three 5-mm trocars) were positioned concentrically around the left liver lobe.

Patients were placed in the supine position with the legs apart. The main surgeon stood on the patient's right side, the assistant surgeon on the left, and the scopist between the patient's legs. After general anesthesia, the first trocar was inserted through a 20-mm incision made on the top of the navel. The pneumoperitoneum was created with the Hasson method and intraabdominal pressure was maintained at 10 mmHg. Four operating trocars were inserted concentrically around the left liver lobe (Figure 1).

**Figure 2:**
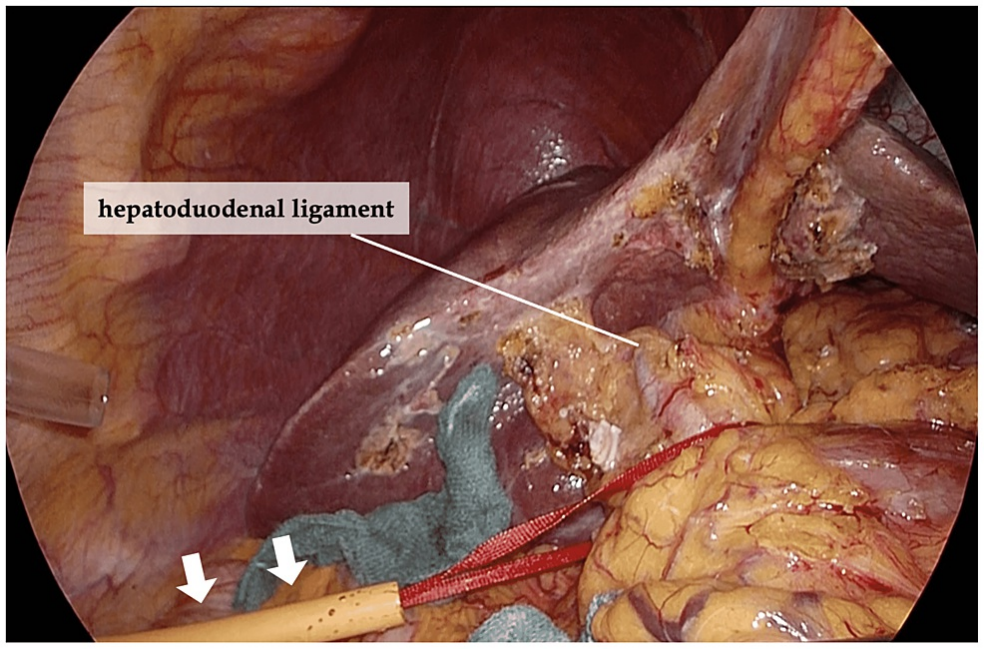
Extraperitoneal Pringle maneuver The extraperitoneal tourniquet system for intermittent vascular occlusion of the hepatoduodenal ligament (Pringle maneuver) was placed below the right costal margin (arrows).

The extraperitoneal tourniquet system for intermittent vascular occlusion of the hepatoduodenal ligament (Pringle maneuver) was placed below the right costal margin (Figures 1-2) [10].

The Pringle maneuver was selectively applied in the case of bleeding during liver parenchymal transection and transecting the left portal pedicle with a laparoscopic vascular stapler.

**Figure 3:**
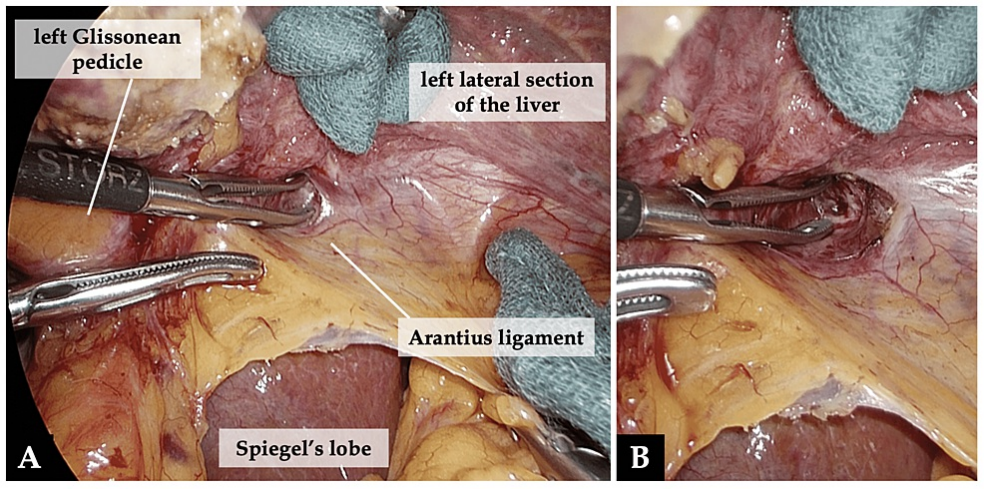
Isolation of the left Glissonean pedicle (part 1). A: Lifting the left lateral section of the liver, the liver parenchyma behind the left Glissonean pedicle was dissected at the ventral aspect of the Arantius ligament. B: The outlet orifice for the subsequent left Glissonean pedicle isolation.

After the gallbladder was removed, the following procedures were performed: (1) Lifting the left lateral section of the liver, the liver parenchyma behind the left Glissonean pedicle was dissected at the ventral aspect of the Arantius ligament to make the outlet orifice for the subsequent left Glissonean pedicle isolation (Figure 3) [7,11].

**Figure 4:**
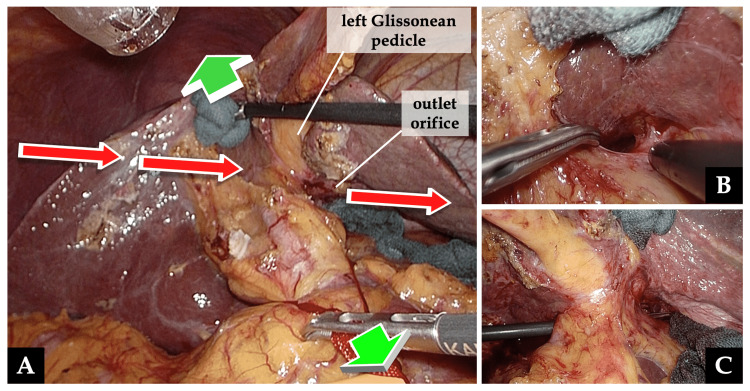
Isolation of the left Glissonean pedicle (part 2) A, C: The left Glissonean pedicle isolation toward the outlet orifice in Figure 3. The assistant surgeon pushed Segment 4 of the liver cranially and the tape encircling the hepatoduodenal ligament caudally in the opposite ways (green arrows), orthogonal to the direction of the laparoscopic forceps toward the left portal triad (red arrows). B: Entry hole cranial to the hilum for the left Glissonean pedicle isolation.

**Figure 5:**
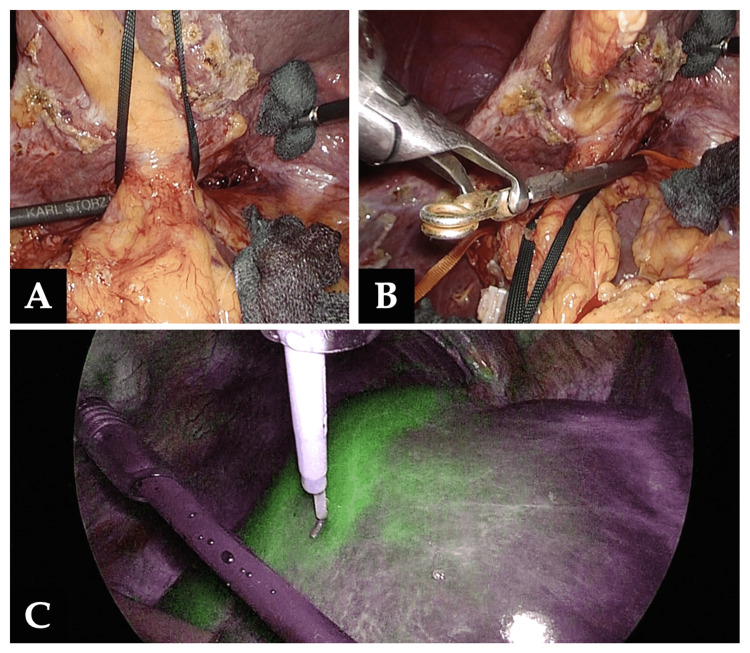
Clamp of the left Glissonean pedicle A, B: The left Glissonean pedicle was taped and clamped by a temporary vascular clamp to visualize the ischemic range of the left liver lobe. C: Intraoperative indocyanine green (ICG) fluorescence for negative staining was used to identify the interlobular plane more accurately.

**Figure 6:**
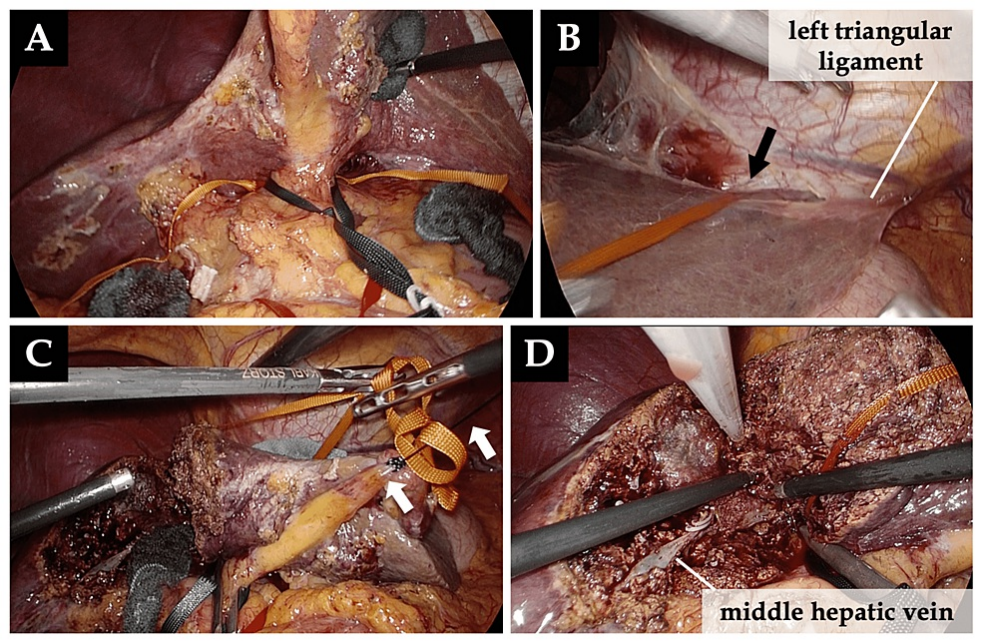
Hanging maneuver for the liver parenchymal transection A, B: Hanging tape (orange tape) across the left lobe of the liver passed behind the left Glissonean pedicle and through the left coronary ligament (arrow). The left triangular ligament was preserved in order to fix the left hemiliver. C: The round ligament was ligated and pulled extracorporeally toward the left costal margin (arrows). D: Liver parenchymal dissection was performed with the wide and stable transection plane.

(2) The left Glissonean pedicle was isolated from the cranial to the hilum toward the aforementioned outlet orifice. At this time, the assistant surgeon pushed Segment 4 of the liver cranially and pulled the tape encircling the hepatoduodenal ligament caudally in the opposite direction to make the laparoscopic forceps easier to encircle the left portal triad with satisfactory traction (Figure 4).

(3) The left Glissonean pedicle was clamped by a temporary vascular clamp to visualize the ischemic range of the left liver lobe. Intraoperative indocyanine green (ICG) fluorescence for negative staining was also used to identify the interlobular plane more accurately (Figure 5).

(4) The round ligament, falciform ligament, and left coronary ligament were transected with an ultrasonic device. The round ligament was ligated and pulled extracorporeally toward the left costal margin during liver parenchymal transection (Figure 1 and Figure 6C).

The left triangular ligament was preserved until the end of parenchymal transection in order to stabilize the left hemiliver (Figure 6B).

**Figure 7:**
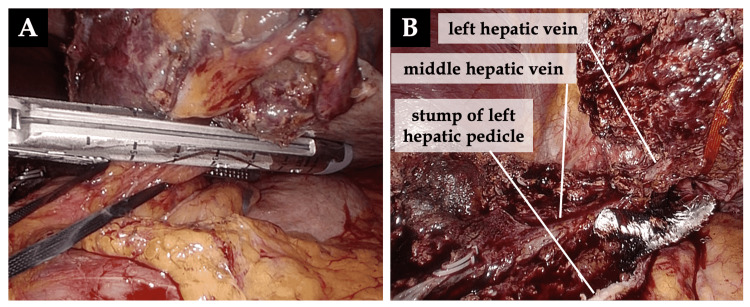
Transection of the left Glissonean pedicle and the left hepatic vein A: The left Glissonean pedicle was transected using a vascular stapler ventral to the Arantius ligament. B: The left hepatic vein to be transected.

(5) Liver parenchymal transection was performed using a Cavitron ultrasonic surgical aspirator (CUSA; Integra Lifesciences Corporation, NJ) and an ultrasonic device. The assistant surgeon pulls the hanging tape across the left liver lobe on the ventral aspect of the Arantius ligament in order to provide a wide and stable liver transection plane (Figure 6).

(6) The left Glissonean pedicle was transected using a vascular stapler ventral to the Arantius ligament [4,6,12], followed by stapling of the left hepatic vein (Figure 7).

## Results

A total of nine patients underwent our standardized laparoscopic left hemihepatectomy without Spiegel’s lobe resection over the last two years in our department (Table 1).

**Table 1:** Patient characteristics BMI: body mass index, HCC: hepatocellular carcinoma

Case No.	Age (years)	Sex	BMI (kg/m^2^)	Pathologic condition	Operative time (min)	Estimated blood loss (ml)	Postoperative hospital stay (days)
1	70	F	26.1	HCC	337	50	8
2	60	M	26.8	colorectal liver metastasis	267	54	9
3	58	M	22.3	colorectal liver metastasis	310	100	11
4	74	M	26.1	colorectal liver metastasis	393	375	8
5	70	M	22.5	colorectal liver metastasis	219	100	16
6	84	M	26.6	HCC	478	170	10
7	72	M	22.6	hepaticolithiasis	305	250	8
8	69	M	16.2	inflammatory pseudotumor	403	232	13
9	75	F	29.4	colorectal liver metastasis	343	41	7

The median age of the nine patients was 70 years (range: 58 - 84 years). Most of the patients were males (77.8%). Five of nine patients were diagnosed with colorectal liver metastasis, two with hepatocellular carcinoma (HCC), one with inflammatory pseudotumor, and the other one with hepaticolithiasis. There were no conversions to open surgery. The median operative time and estimated blood loss were 337 minutes (range: 219 - 478 minutes) and 100 ml (range: 41 - 375 ml), respectively. The median length of postoperative hospital stay was nine days (range: 7 - 16 days). Major complications (Clavien-Dindo classification grade III or more) were not encountered in our cohort postoperatively.

For five out of nine patients, the left hepatic pedicle was securely transected with a vascular stapler through the umbilical trocar due to the short distance between the left portal pedicle and 12-mm trocar.

A preoperative imaging study revealed that one patient (Case 7 of Table 1) had a Huang type A3 anatomical variation of the biliary tree; the right posterior hepatic duct drained into the left main duct [13]. To confirm whether our procedure stapling the left Glissonean pedicle above the Arantius ligament was safe enough for this type of biliary tract variant, intraoperative cholangiography was performed via an endoscopic nasobiliary drainage (ENBD) tube that was placed preoperatively after endoscopic removal of intrahepatic bile duct stones inside the left main hepatic duct.

**Figure 8:**
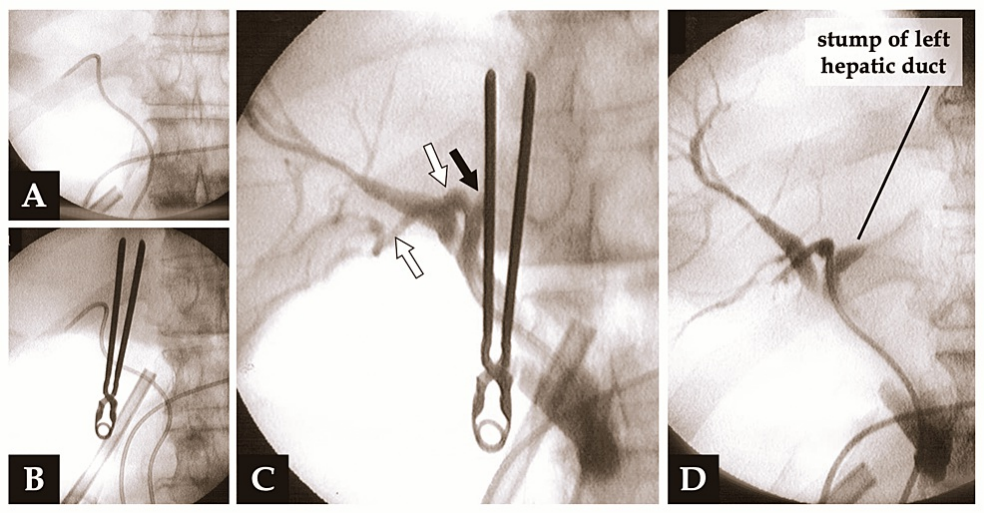
Intraoperative cholangiography of the patient with a Huang type A3 biliary tree variation A: An endoscopic nasobiliary drainage (ENBD) tube was placed in the right posterior bile duct. B: The temporary vascular clamp on the left Glissonean pedicle. C: Intraoperative cholangiography via an ENBD tube. The vascular clamp on the left hepatic pedicle was sufficiently apart from where the right posterior bile duct (white arrows) drained into the left bile duct (black arrow). D: Intraoperative cholangiography after transecting the left Glissonean pedicle.

Figure 8 showed that the temporary vascular clamp on the left hepatic pedicle was sufficiently apart from where the posterior bile duct drained into the left bile duct.

## Discussion

In order to improve the safety and feasibility of laparoscopic left hemihepatectomy without Spiegel’s lobe resection, we clarified the definite assistant's roles when standardizing the operative procedures. In our approach, we make full use of umbilical tapes to overcome the limited number of working trocars. During left hemihepatectomy, assistant support is critical, especially when isolating the left Glissonean pedicle and transecting the liver parenchyma.

For anatomical liver resection, there are two approaches to inflow control: the Glissonean approach and the conventional hilar dissection approach. The Glissonean approach was introduced more than three decades ago [14-15], and recently applied to laparoscopic surgery [16], with the advance in understanding the anatomy of Laennec's capsule of the liver [17].

The Glissonean approach exhibited shorter operative time, less blood loss, and fewer complications compared to the conventional hilar approach in laparoscopic surgeries [18]. Therefore, we generally choose the Glissonean approach for laparoscopic anatomical liver resection.

To efficiently generate countertraction to the hilum when securing the left hepatic pedicle using the Glissonean approach, we propose the technique of pushing Segment 4 of the liver cranially and pulling the tape encircling the hepatoduodenal ligament caudally in the opposite way, which was orthogonal to the direction of the laparoscopic forceps toward the left portal triad. Instead of pushing Segment 4 of the liver, pushing the round ligament in the same direction is also effective.

When transecting liver parenchyma, we employ the hanging tape across the left liver lobe on the ventral side of the Arantius ligament in order to provide a wide and stable liver transection plane. If the hanging tape is hooked on the edge of the liver transection plane, the field of view will be better (Figure 6D).

It is noted that we preserve the round ligament until just before transecting liver parenchyma because dividing it makes the left liver lobe unstabilized, and one of the assistant hands is required to fix it. Although it is mostly possible to encircle the left Glissonean pedicle without transecting the round ligament, we cut it to improve the flexibility of the liver when it is difficult due to an inappropriate forceps angle.

One of the most serious complications of left hemihepatectomy is the stricture of the bile duct to be preserved, especially in the case of a Huang type A3 biliary tree variation; the right posterior hepatic duct drains into the left main duct [13]. Approximately 10% and up to 30% of individuals are reported to have Huang type A3 bile duct anatomy [19-22]. For this variation, some hepatobiliary surgeons consider that the conventional hilar dissection approach is preferable to avoid biliary injury. Because this procedure is slightly complicated and time-consuming, especially in laparoscopic surgeries [18], we tried to verify the safety of the Glissonean approach for a Huang type A3 biliary variation using intraoperative cholangiography. In our series, one patient had this type of bile duct variant, and the patency of the right posterior bile duct, which was inserted into the left hepatic duct, was secured by stapling the left Glissonean pedicle ventral to the Arantius ligament [4,6,12].

Previous studies reported that dividing the Arantius ligament made it easier to encircle the left hepatic pedicle and left hepatic vein [3,18,23]. However, this technique may cause stapling of the left Glissonean pedicle dorsal to the Arantius ligament and there is a concern that the staple may involve the convergence of the right posterior and left hepatic ducts.

Our study does have limitations. First, the sample size is small due to the short study period after the standardization of the surgical procedures. Second, intraoperative cholangiography was performed in only one patient with a Huang type A3 biliary tree variation. If bile duct injury caused by stapling needs to be thoroughly evaluated, intraoperative cholangiography should be carried out in all cases. In our cohort, no cases of bile duct stricture were found during postoperative follow-up.

In order to complete left hemihepatectomy without Spiegel’s lobe resection in a safe manner, it is recommended to preserve the Arantius ligament followed by transection of the left Glissonean pedicle at the ventral aspect of it.

## Conclusions

We presented here our standardized assistant roles during laparoscopic left hemihepatectomy without Spiegel’s lobe resection. By clarifying the roles of the assistant surgeon, the laparoscopic approach will become more feasible for left hemihepatectomy. While ensuring the quality of surgery, we would like to connect it to the education of young surgeons who learn laparoscopic anatomical hepatectomy.
